# Mediating role of the systemic immune-inflammation index in obesity-induced glycolipid dysmetabolism and compromised IVF/ICSI outcomes in polycystic ovary syndrome: A retrospective cohort study

**DOI:** 10.1097/MD.0000000000048005

**Published:** 2026-03-20

**Authors:** Xiaofang Zhang, Ling Lin, Hongshan Ge

**Affiliations:** a Reproduction Medicine Center, Taizhou People’s Hospital affiliated with Nanjing Medical University, Taizhou, China.

**Keywords:** insulin resistance, IVF/ICSI outcomes, lipid ratios, polycystic ovary syndrome, systemic immune-inflammation index

## Abstract

This study aims to investigate the mediating role of the systemic immune-inflammation index (SII) in the relationship between obesity-related glycolipid indices and in vitro fertilization/intracytoplasmic sperm injection (IVF/ICSI) outcomes in women with polycystic ovary syndrome (PCOS). A total of 598 women diagnosed with PCOS according to the Rotterdam criteria and undergoing their first IVF/ICSI cycle at the Reproduction Medicine Center, Taizhou People’s Hospital Affiliated with Nanjing Medical University, Jiangsu, China between January 2021 and December 2023 were included. Key exposures included obesity-related metabolic indices (e.g., triglyceride to high-density lipoprotein ratio [TG/HDL], homeostasis model assessment of insulin resistance [HOMA-IR]) and the SII. The primary outcome was the live birth rate per initiated cycle. Associations were evaluated using multivariate generalized linear models, and causal mediation analysis was performed to quantify the proportion of the effect mediated by the SII. Higher TG/HDL, total cholesterol to HDL ratio (TC/HDL), low-density lipoprotein to HDL ratio (LDL/HDL), and HOMA-IR levels showed dose-dependent negative correlations with oocyte yield, fertilization rate, embryo quality, and live birth rate (all *P* < .05). An elevated SII was an independent predictor of a reduced live birth rate (β = −0.08, *P* = .008) and mediated 8.8% to 10.7% of the adverse effects of dyslipidemia (via TC/HDL and LDL/HDL) on live birth. This study shows that the SII is statistically linked to and potentially mediates the connection between metabolic dysfunction and poor IVF/ICSI outcomes in PCOS. Integrated strategies targeting both metabolism and inflammation may optimize fertility success in this population.

## 1. Introduction

Polycystic ovary syndrome (PCOS) is a common endocrine and metabolic disorder characterized by a constellation of clinical features, primarily including oligo-anovulation, clinical or biochemical hyperandrogenism, and polycystic ovarian morphology. It represents one of the most prevalent endocrine conditions among women of reproductive age, affecting an estimated 4% to 21% of this population globally, with variation depending on the diagnostic criteria applied and the population studied.^[1-3]^ Beyond reproductive dysfunction, PCOS confers substantial metabolic risks, including insulin resistance (IR), dyslipidemia, and obesity (prevalence: 38–88%),^[4-6]^ which negatively impact the efficacy of medically assisted reproductive treatments^[7-16]^ and independently reduce in vitro fertilization/intracytoplasmic sperm injection (IVF/ICSI) live birth rates by 20% to 30%.^[17,18]^

Polycystic ovary syndrome is underpinned by chronic low-grade inflammation driven by adipose tissue dysfunction and metabolic dysregulation.^[19,20]^ While conventional markers like C-reactive protein offer limited prognostic specificity, the systemic immune-inflammation index (SII = platelets × neutrophils/lymphocytes) provides a unique, integrative measure of 3 key inflammatory axes: neutrophil-mediated tissue inflammation, lymphocyte-regulated immune homeostasis, and platelet-associated thrombotic activity.^[21]^ This composite design grants SII superior sensitivity in capturing the multidimensional inflammatory milieu of PCOS, reflecting critical crosstalk between metabolic stress and immune activation.^[22-25]^ Mechanistically, obesity and dyslipidemia activate NLRP3 inflammasomes and NF-κB signaling,^[26]^ elevating pro-inflammatory cytokines (e.g., interleukin-6 [IL-6], tumor necrosis factor-α [TNF-α]) that impair folliculogenesis and endometrial receptivity.^[27]^ Critically, SII leverages routinely available complete blood count parameters, offering a pragmatic, cost-effective biomarker for clinical risk stratification in reproductive medicine – a distinct advantage over specialized assays.^[21]^

Despite established links between metabolic dysfunction and IVF/ICSI failure, SII’s role as a causal mediator in the metabolic-reproductive axis remains unexplored. We hypothesize that SII integrates obesity-related metabolic insults into a unified inflammatory response that directly compromises IVF/ICSI success. This study investigates: Associations between obesity/dyslipidemia and IVF/ICSI outcomes; SII as an independent predictor of IVF/ICSI failure; and SII’s mechanistic mediation of metabolic-reproductive dysfunction in PCOS.

## 2. Materials and methods

### 2.1. Ethical statement

Approval for this study was obtained from the institutional review board of Taizhou People’s Hospital affiliated with Nanjing Medical University (No. KY2023-117-01). Written informed consent was obtained from all participants.

### 2.2. Study population

We screened 598 women with PCOS (aged 20–45) who underwent their first autologous IVF/ICSI cycles at our center between January 2021 and December 2023. Polycystic ovary syndrome was diagnosed according to the revised Rotterdam criteria, requiring at least 2 of the following: clinical or biochemical hyperandrogenism; oligo- or anovulation; polycystic ovaries on ultrasound.

Exclusion criteria included uterine abnormalities; adrenal, thyroid, or metabolic disorders; autoimmune diseases; recurrent pregnancy loss; chromosomal abnormalities in either spouse; or participation in in vitro maturation or preimplantation genetic testing cycles.

### 2.3. IVF/ICSI procedures

We followed standardized controlled ovarian hyperstimulation protocols, with gonadotropin dosing individualized based on patient age, serum anti-Müllerian hormone (AMH) levels, antral follicle count, and body mass index (BMI). Oocyte retrieval, insemination, and embryo culture were performed per institutional protocols. Fertilization was assessed on day 1, with embryo morphology evaluated on day 3 and day 5/6. For frozen-thawed embryo transfers, natural cycles were used for ovulatory women, while programmed cycles were employed for anovulatory patients or those requesting hormonal regulation. A maximum of 2 day 3 embryos or day 5/6 blastocysts were transferred by experienced reproductive specialists.

### 2.4. Laboratory assessment

Serum total triglyceride (TG), total cholesterol (TC), high-density lipoprotein cholesterol (HDL), low-density lipoprotein cholesterol (LDL), fasting plasma glucose (FPG) and fasting insulin (FINS) were measured during 2 months before ovarian stimulation. Peripheral blood platelet, neutrophil, lymphocyte counts was determined within 1 week before ovarian stimulation.

### 2.5. Study outcomes

The study outcomes included: serum peak estradiol (E_2_) level, serum luteinizing hormone (LH) level, serum progestin (P) level on the ovulatory triggering day, retrieved oocyte counts, metaphase II (MII) oocyte counts, normally fertilized zygote counts, normally cleaved embryo counts, high quality day 3 embryo counts, blastocyst formation counts, high quality blastocyst counts, biochemical pregnancy, clinical pregnancy, and live birth.

### 2.6. Statistical analysis

All analyses were conducted using “R” software (version 4.4.2; R Core Team, The R Foundation for Statistical Computing, Vienna, Austria, https://www.r-project.org). Baseline characteristics were summarized as median [IQR] or n (%) and compared across BMI categories using Kruskal–Wallis tests for continuous variables and chi-squared/Fisher exact tests for categorical variables.

Multivariate generalized linear models were employed to evaluate: associations between obesity/glucolipid metabolism indicators and IVF/ICSI outcomes and the effect of SII on treatment outcomes in PCOS women. Potential confounders including age, AMH, antral follicle count (AFC), basal follicle-stimulating hormone (FSH), infertility type and duration, stimulation protocol, insemination technique, embryo transfer timing, and embryo quality were considered. Covariates remained in final models if they altered effect estimates by >10%. Statistical significance was set at *P* < .05.

## 3. Results

### 3.1. Baseline reproductive and cycle characteristics

As BMI class increased, participants were significantly older (*P* < .01) and exhibited higher AFC (*P* < .05) alongside lower basal FSH levels (*P* < .01). Concurrently, higher BMI was associated with elevated levels of FPG, FINS, HOMA-IR, and SII, as well as decreased HDL (all *P* < .001). Significant differences were also observed in infertility type and duration across the BMI groups (*P* = .019 and *P* = .022, respectively). These findings indicate that increasing BMI in PCOS patients is associated with progressive alterations in reproductive, metabolic, and inflammatory profiles (Table 1).

**Table 1:** **Baseline reproductive and cycle characteristics of the PCOS women undergoing the first IVF/ICSI cycles**.

Characteristics	BMI (kg/m^2^)				
	<18.5	18.5–25	25–30	≥30	*P* *
n = 598	n = 32	n = 341	n = 148	n = 77	
Age (yr)	27.50 [26.00, 29.00]	30.00 [27.00, 33.00]	30.00 [27.00, 33.00]	29.00 [27.00, 31.00]	.001
AMH (ng/mL)	6.13 [4.33, 8.85]	5.57 [3.95, 8.33]	5.50 [3.88, 7.80]	5.20 [3.81, 8.77]	.857
AFC (n)	12.00 [9.88, 12.00]	12.00 [10.00, 12.00]	12.00 [11.00, 12.00]	12.00 [11.00, 12.00]	.048
Basal FSH (mIU/mL)	6.68 [5.63, 7.84]	6.04 [4.92, 7.14]	5.53 [4.85, 6.75]	5.32 [4.35, 6.63]	.006
TG (mmol/L)	1.42 [0.86, 1.80]	1.27 [0.92, 2.00]	1.44 [0.97, 2.35]	1.36 [1.06, 1.96]	.201
TC (mmol/L)	4.83 [4.09, 5.58]	4.80 [4.26, 5.44]	4.73 [4.00, 5.58]	4.94 [4.33, 5.61]	.678
HDL (mmol/L)	1.30 [1.14, 1.64]	1.38 [1.19, 1.62]	1.29 [1.11, 1.54]	1.24 [1.07, 1.45]	.001
LDL (mmol/L)	3.07 [2.58, 3.48]	2.99 [2.61, 3.46]	3.06 [2.49, 3.52]	3.13 [2.70, 3.66]	.387
FPG (mmol/L)	4.94 [4.43, 5.19]	4.95 [4.60, 5.32]	5.11 [4.78, 5.50]	5.32 [4.84, 5.83]	<.001
FINS (pmol/L)	62.10 [50.02, 83.12]	69.40 [50.70, 102.60]	84.81 [60.05, 117.08]	124.60 [93.50, 200.90]	<.001
HOMA-IR	1.94 [1.59, 2.54]	2.19 [1.59, 3.26]	2.72 [1.86, 3.90]	4.36 [2.97, 6.97]	<.001
SII (10^9^/L)	462.63 [337.85, 669.25]	450.59 [342.84, 605.90]	485.15 [355.04, 682.09]	611.96 [454.76, 880.24]	<.001
Infertility type					.019
Primary	16 (50.0)	198 (58.1)	50 (33.8)	52 (74.0)	
Secondary	16 (50.0)	143 (41.9)	50 (33.8)	20 (26.0)	
Duration of infertility (yr)	2.00 [1.00, 3.25]	3.00 [2.00, 4.00]	3.00 [2.00, 4.00]	3.00 [2.00, 5.00]	.022
Insemination technique					.543
IVF	16 (81.2)	272 (79.8)	126 (85.1)	61 (79.2)	
ICSI	6 (18.8)	69 (20.2)	22 (14.9)	16 (20.8)	
Ovarian stimulation regimen					.407
Long GnRH-a	15 (46.9)	127 (37.2)	63 (42.6)	34 (44.2)	
GnRH antagonist	10 (31.2)	109 (32.0)	37 (25.0)	16 (20.8)	
Minimal stimulation protocol	6 (18.8)	91 (26.7)	44 (29.7)	26 (33.8)	
Others†	1 (3.1)	14 (4.1)	4 (2.7)	1 (1.3)	
Transplantation program					.222
Natural cycle	24 (75.0)	228 (66.9)	101 (68.2)	53 (68.8)	
Hormone replacement	0 (0.0)	52 (15.2)	15 (10.1)	9 (11.7)	
Down-regulate + hormone replacement	8 (25.0)	61 (17.9)	32 (21.6)	15 (19.5)	
Timing of embryo transfer					.083
Day 3	18 (56.2)	161 (47.2)	83 (56.1)	41 (53.2)	
Day 5	13 (40.6)	172 (50.4)	61 (41.2)	30 (39.0)	
Day 6	1 (3.1)	8 (2.3)	4 (2.7)	6 (7.8)	
Transferred embryos quantity					.658
Low	13 (40.6)	113 (33.1)	444 (29.7)	24 (31.2)	
High	19 (59.4)	228 (66.9)	104 (70.3)	53 (68.8)	

AFC = antral follicle count, AMH = anti-Müllerian hormone, BMI = body mass index, FINS = fasting insulin, FPG = fasting plasma glucose, FSH = follicle-stimulating hormone, GnRH = gonadotropin-releasing hormone, HDL = high-density lipoprotein cholesterol, HOMA-IR = homeostasis model assessment of insulin resistance, ICSI = intracytoplasmic sperm injection, IVF = in vitro fertilization, LDL = low-density lipoprotein cholesterol, PCOS = polycystic ovary syndrome, SII = systemic immune-inflammation index, TC = total cholesterol, TG = triglycerides.

* *P*-values comparing the differences across the 4 BMI groups.

† Other protocols include the short GnRH-a protocol and ultrashort GnRH antagonist protocol.

### 3.2. Obesity and associated metabolism indicators and IVF/ICSI outcomes in PCOS women

We observed significant inverse associations between multiple metabolic indices (TG/HDL, TC/HDL, LDL/HDL, HOMA-IR) and early embryological outcomes. All 4 indices showed negative correlations with normal cleavage rates, total oocyte yield, MII oocyte count, and normally fertilized zygote counts (all *P* < .001).

Specifically, TG/HDL and HOMA-IR were negatively associated with high-quality day 3 embryos and blastocyst formation rates (all *P* < .001), while TC/HDL and LDL/HDL also demonstrated significant inverse relationships with these parameters (all *P* < .05). These findings highlight metabolic dysregulation as a potential modifiable factor affecting IVF/ICSI success in obese PCOS women (Tables 2–5).

**Table 2:** **β (95% CI) in hormone levels on the ovulatory triggering day associated with obesity and associated metabolism indicators among PCOS women undergoing their first IVF/ICSI cycles based on generalized linear models (n = 598**).

Obesity and associated metabolism indicators	Peak E_2_ levels, pmol/L		LH levels, mIU/mL		P levels, nmol/L	
	β (95% CI)	*P*	β (95% CI)	*P*	β (95% CI)	*P*
BMI, kg/m^2^	−311.24 (−462.83, −159.64)	<.001	0.01 (−0.05, 0.06)	.90	−0.05 (−0.09, −0.01)	.04
TG/HDL	−1189.07 (−1907.46, −470.67)	.001	0.10 (−0.16, 0.35)	.46	−0.12 (−0.33, 0.10)	.29
TC/HDL	−76.87 (−816.03, 662.29)	.84	0.04 (−0.22, 0.30)	.77	0.01 (−0.20, 0.23)	.91
LDL/HDL	−355.54 (−1360.44, 649.36)	.49	0.10 (−0.26, 0.46)	.58	0.02 (−0.27, 0.31)	.89
HOMA-IR	−297.95 (−590.02, −5.88)	.046	−0.03 (−0.14, 0.07)	.56	−0.01 (−0.09, 0.08)	.91

BMI = body mass index, CI = confidence interval, E2 = estradiol, HDL = high-density lipoprotein cholesterol, HOMA-IR = homeostasis model assessment of insulin resistance, ICSI = intracytoplasmic sperm injection, IVF = in vitro fertilization, LDL = low-density lipoprotein cholesterol, LH = luteinizing hormone, P = progesterone, PCOS = polycystic ovary syndrome, TC = total cholesterol, TG = triglycerides.

**Table 3:** **RR (95% CI) in oocyte development outcomes, and fertilization associated with obesity and associated metabolism indicators among PCOS women undergoing their first IVF/ICSI cycles based on generalized linear models (n = 598**).

Obesity and associated metabolism indicators	Retrieved oocytes, n		MII oocytes, n		Normal fertilization, n	
	RR (95% CI)	*P*	RR (95% CI)	*P*	RR (95% CI)	*P*
BMI, kg/m^2^	1.00 (0.99, 1.00)	.043	1.00 (0.99, 1.00)	.15	0.99 (0.99, 1.00)	.06
TG/HDL	0.78 (0.76, 0.80)	<.001	0.77 (0.74, 0.79)	<.001	0.77 (0.74, 0.80)	<.001
TC/HDL	0.93 (0.91, 0.96)	<.001	0.94 (0.92, 0.97)	<.001	0.92 (0.89, 0.95)	<.001
LDL/HDL	0.88 (0.86, 0.91)	<.001	0.89 (0.86, 0.92)	<.001	0.86 (0.83, 0.90)	<.001
HOMA-IR	0.97 (0.96, 0.98)	<.001	0.96 (0.95, 0.97)	<.001	0.96 (0.95, 0.97)	<.001

CI = confidence interval, BMI = body mass index, HDL = high-density lipoprotein cholesterol, HOMA-IR = homeostasis model assessment of insulin resistance, ICSI = intracytoplasmic sperm injection, IVF = in vitro fertilization, LDL = low-density lipoprotein cholesterol, MII = metaphase II, PCOS = polycystic ovary syndrome, RR = risk ratio, TC = total cholesterol, TG = triglycerides.

**Table 4:** **RR (95% CI) in early embryo development outcomes associated with obesity and associated metabolism indicators among PCOS women undergoing their first IVF/ICSI cycles based on generalized linear models (n = 598**).

Obesity and associated metabolism indicators	Normal cleavage, n		High-quality day 3 embryos, n		Blastocyst formation, n		High-quality blastocyst, n	
	RR (95% CI)	*P*	RR (95% CI)	*P*	RR (95% CI)	*P*	RR (95% CI)	*P*
BMI, kg/m^2^	0.99 (0.99, 1.00)	.038	0.99 (0.98, 1.00)	.097	0.99 (0.98, 1.00)	.052	0.98 (0.97, 0.99)	.008
TG/HDL	0.77 (0.74, 0.80)	<.001	0.83 (0.79, 0.88)	<.001	0.86 (0.81, 0.92)	<.001	0.87 (0.79, 0.94)	<.001
TC/HDL	0.92 (0.89, 0.95)	<.001	0.95 (0.91, 0.99)	.029	0.92 (0.88, 0.97)	.003	0.86 (0.80, 0.92)	<.001
LDL/HDL	0.86 (0.82, 0.90)	<.001	0.91 (0.85, 0.96)	.002	0.90 (0.83, 0.96)	.002	0.85 (0.77, 0.93)	<.001
HOMA-IR	0.96 (0.95, 0.97)	<.001	0.95 (0.93, 0.97)	<.001	0.96 (0.94, 0.98)	<.001	0.96 (0.93, 0.99)	.01

CI = confidence interval, BMI = body mass index, HDL = high-density lipoprotein cholesterol, HOMA-IR = homeostasis model assessment of insulin resistance, ICSI = intracytoplasmic sperm injection, IVF = in vitro fertilization, LDL = low-density lipoprotein cholesterol, PCOS = polycystic ovary syndrome, RR = risk ratio, TC = total cholesterol, TG = triglycerides.

**Table 5:** **OR (95% CI) in pregnancy outcomes associated with obesity and associated metabolism indicators among PCOS women undergoing their first IVF/ICSI cycles based on generalized linear models (n = 598**).

Obesity and associated metabolism indicators	Biochemical pregnancy		Clinical pregnancy		Live birth	
	OR (95% CI)	*P*	OR (95% CI)	*P*	OR (95% CI)	*P*
BMI, kg/m^2^	1.04 (1.00, 1.10)	.08	1.02 (0.98, 1.07)	.36	0.98 (0.94, 1.02)	.31
TG/HDL	0.85 (0.70, 1.05)	.12	0.84 (0.70, 1.02)	.07	0.84 (0.70, 1.01)	.06
TC/HDL	0.90 (0.72, 1.12)	.36	0.91 (0.74, 1.12)	.36	0.93 (0.77, 1.12)	.43
LDL/HDL	0.81 (0.60, 1.09)	.16	0.79 (0.60, 1.04)	.09	0.82 (0.64, 1.06)	.13
HOMA-IR	0.96 (0.88, 1.04)	.29	0.95 (0.88, 1.03)	.20	0.96 (0.89, 1.03)	.26

CI = confidence interval, BMI = body mass index, HDL = high-density lipoprotein cholesterol, HOMA-IR = homeostasis model assessment of insulin resistance, ICSI = intracytoplasmic sperm injection, IVF = in vitro fertilization, LDL = low-density lipoprotein cholesterol, OR = odds ratio, PCOS = polycystic ovary syndrome, TC = total cholesterol, TG = triglycerides.

### 3.3. SII and IVF/ICSI outcomes in PCOS women

After controlling for FSH, AFC, and ovarian stimulation regimen, we found increasing SII was significantly associated with decreasing live birth rates (*P* = .008; Table 6), and it was associated with decreasing retrieved oocytes count, MII oocytes count, normally fertilized zygote count, normally cleaved embryo count, blastocyst formation count and clinical pregnancy rates (all *P* < .05). Embodied systemic inflammation (measured by SII) adversely affects IVF/ICSI outcomes in PCOS.

**Table 6:** **β/RR/OR (95% CI) in reproductive and pregnancy outcomes associated with SII among PCOS women undergoing their first IVF/ICSI cycles based on generalized linear models (n = 598**).

Outcomes	SII, ×10^9^/L	
	β/RR/OR (95% CI)	*P*
Peak E2 levels, pmol/L	−30.48 (−278.50, 217.54)	.81
LH levels, mIU/ml	0.08 (−0.01, 0.16)	.08
P levels, nmol/L	−0.02 (−0.09, 0.06)	.66
Retrieved oocytes, n	0.99 (0.98, 1.00)	.02
MII oocytes, n	0.99 (0.98, 1.00)	.01
Normal fertilization, n	0.99 (0.98, 1.00)	.04
Normal cleavage, n	0.99 (0.98, 1.00)	.02
High-quality day 3 embryos, n	0.99 (0.97, 1.00)	.07
Blastocyst formation, n	0.98 (0.96, 1.00)	.03
High-quality blastocysts, n	0.99 (0.96, 1.01)	.38
Biochemical pregnancy	0.9l4 (0.88, 1.01)	.07
Clinical pregnancy	0.93 (0.87, 0.99)	.02
Live birth	0.92 (0.86, 0.98)	.008

Adjusted for FSH (continuous), AFC (continuous), and ovarian stimulation regimen.

AFC = antral follicle count, CI = confidence interval, FSH = follicle-stimulating hormone, ICSI = intracytoplasmic sperm injection, IVF = in vitro fertilization, LH = luteinizing hormone, MII = metaphase II, OR = odds ratio, P = progesterone, PCOS = polycystic ovary syndrome, RR = risk ratio, SII = systemic immune-inflammation index.

### 3.4. SII counts and obesity and associated metabolism indicators in PCOS women

After controlling for FSH, AFC, and ovarian stimulation regimen, BMI and HOMA-IR were positively associated with SII (both *P* < .001). TG/HDL, TC/HDL and LDL/HDL were associated with SII (all *P *< .05). Systemic immune-inflammation index may act as a functional integrator of metabolic dysfunction (obesity, IR, dyslipidemia) in PCOS (Table 7).

**Table 7:** **β (95% CI) in SII associated with obesity and associated metabolism indicators among PCOS women undergoing their first IVF/ICSI cycles based on generalized linear models (n = 598**).

Obesity and associated metabolism indicators	β (95% CI)	*P*
BMI, kg/m^2^	14.05 (9.12, 18.98)	<.001
TG/HDL	22.84 (2.31, 43.37)	.029
TC/HDL	40.22 (16.09, 64.35)	.001
LDL/HDL	53.67 (20.84, 86.50)	.001
HOMA-IR	27.43 (18.04, 36.82)	<.001

Adjusted for FSH (continuous), AFC (continuous), and ovarian stimulation regimen.

AFC = antral follicle count, BMI = body mass index, CI = confidence interval, FSH = follicle-stimulating hormone, HDL = high-density lipoprotein cholesterol, HOMA-IR = homeostasis model assessment of insulin resistance, ICSI = intracytoplasmic sperm injection, IVF = in vitro fertilization, LDL = low-density lipoprotein cholesterol, PCOS = polycystic ovary syndrome, SII = systemic immune-inflammation index, TC = total cholesterol, TG = triglycerides.

### 3.5. Mediation analyses

**Figure 1.**
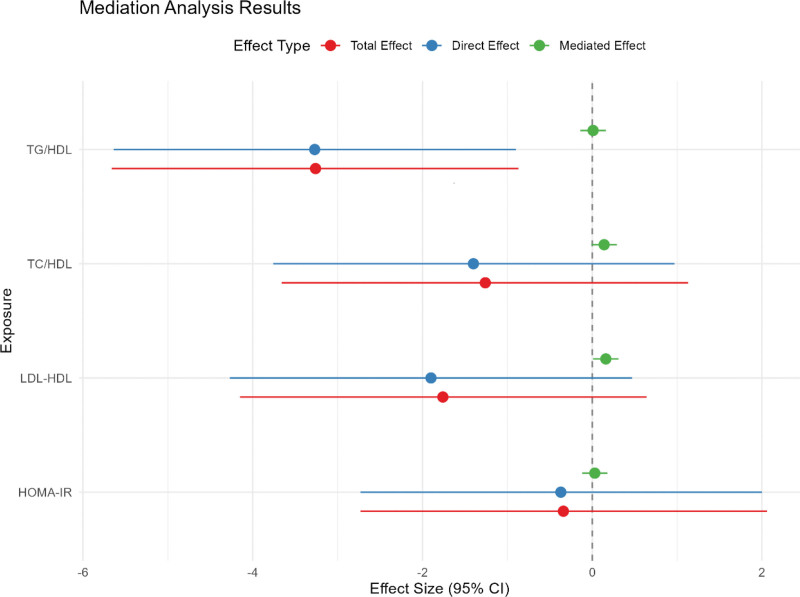
Mediating effects of SII on the associations between obesity and associated metabolism indicators and live birth. CI = confidence interval, HDL = high-density lipoprotein cholesterol, HOMA-IR = homeostasis model assessment of insulin resistance, LDL = low-density lipoprotein cholesterol, SII = systemic immune-inflammation index, TC = total cholesterol, TG = triglycerides.

Considering that SII was associated with obesity and associated metabolic indicators as well as IVF/ICSI outcomes, we hypothesized that SII could be a possible mediator. Table 8 and Figure 1 show the results of the mediation analysis. After controlling for confounders and SII, we found that SII significantly mediated the effect of TC/HDL and LDL/HDL on live birth rates (*P* < .05), with mediation ratios of −10.7% and −8.8%, respectively. Given the mediating effects of SII, interventions targeting metabolic health (weight loss, insulin sensitizers, lipid control) could concurrently reduce inflammation and improve IVF/ICSI outcomes.

**Table 8:** **Mediation analyses investigating whether SII mediated the associations between obesity and associated metabolism indicators and live birth**.

Mediators	Associations	Total effect (95% CI)	Mediated effect (95% CI)	Estimated proportion mediated (%)
SII, ×10^9^/L	Live birth and TG/HDL	−3.26 (−5.66, −0.87)**	0.01 (−0.14, 0.16)	−0.3
	Live birth and TC/HDL	−1.26 (−3.66, 1.13)**	0.14 (−0.01, 0.29)*	−10.7
	Live birth and LDL/HDL	−1.76 (−4.15, 0.64)**	0.16 (0.01, 0.31)*	−8.8
	Live birth and HOMA-IR	−0.34 (−2.73, 2.06)*	0.03 (−0.12, 0.18)	−7.8

Adjusted for FSH (continuous), AFC (continuous), and ovarian stimulation regimen.

AFC = antral follicle count, CI = confidence interval, FSH = follicle-stimulating hormone, HDL = high-density lipoprotein cholesterol, HOMA-IR = homeostasis model assessment of insulin resistance, LDL = low-density lipoprotein cholesterol, SII = systemic immune-inflammation index, TC = total cholesterol, TG = triglycerides.

* *P* < .05.

** *P* < .01.

## 4. Discussion

This study is the first to employ mediation analysis to reveal that the systemic immune-inflammation index (SII) is statistically associated with and may mediate the link between metabolic dysfunction and adverse in vitro fertilization/intracytoplasmic sperm injection (IVF/ICSI) outcomes in women with polycystic ovary syndrome (PCOS). Our findings delineate a coherent pathophysiological hypothetical pathway: obesity-induced insulin resistance and dyslipidemia elevate SII, reflecting a state of chronic inflammation that directly impairs oocyte competence, early embryo development, and ultimately live birth rates. Crucially, mediation analyses revealed that SII mediated 8.8% to 10.7% of the detrimental effects of dyslipidemia (specifically elevated TC/HDL and LDL/HDL ratios) on live birth. This indicates that systemic inflammation, as quantified by the SII, may represent a potential and intervenable nexus within the PCOS metabolic-reproductive axis, with the prospect of being developed into a actionable therapeutic target.

The findings underscore the significant clinical utility of SII in the assessment and management of PCOS patients undergoing IVF/ICSI treatment. SII’s primary strength lies in its derivation from universally available, routine complete blood count parameters (platelet, neutrophil, lymphocyte counts). This makes it highly pragmatic and cost-effective for implementation in diverse clinical settings, including resource-limited environments, unlike specialized assays for cytokines (e.g., IL-6, TNF-α) or adipokines which are often costly and not routinely performed. Its calculation is simple and readily integrable into electronic medical records. Besides, SII provides a unique, integrative measure reflecting the interplay of innate immunity (neutrophils), adaptive immunity (lymphocytes), and coagulation/thrombosis (platelets). This multidimensional view offers a more holistic assessment of the systemic inflammatory state compared to isolated markers like C-reactive protein (primarily hepatic acute phase response) or neutrophil-to-lymphocyte ratio (lacking platelet contribution).^[24,25]^ Our data confirm that SII is significantly correlated with metabolic dysfunction (obesity, insulin resistance, dyslipidemia) in PCOS and can serve as a functional integrative indicator thereof (Table 7). We also demonstrated that elevated SII is a significant independent predictor of reduced oocyte yield, fertilization rates, embryo development (cleavage, blastulation), and critically, clinical pregnancy and live birth rates in PCOS women undergoing IVF/ICSI, even after adjusting for key confounders (Table 6). This preliminarily supports the potential of SII as a prognostic tool for identifying PCOS patients at high risk of IVF/ICSI failure.

Moreover, our data suggest a potential clinically relevant threshold. The median SII in our obese PCOS group (BMI ≥ 25 kg/m^2^), who had significantly worse metabolic profiles, was 611.96 × 10^9^/L. Women with PCOS and SII levels approaching or exceeding this value may represent a high-risk subgroup warranting intensified pre-IVF/ICSI treatment intervention. The statistical mediation effect revealed by SII (Table 8 and Fig. 1) suggests that future interventions should not focus solely on metabolic parameters but also address the potential resultant inflammation. Consequently, SII may guide stratified treatment strategies. For instance, in patients with well-controlled metabolism but persistently high SII, adjuvant anti-inflammatory strategies alongside metabolic optimization may confer additional benefits.^[28]^

Our findings align with and extend the known pathophysiology. Dyslipidemia and IR fuel inflammation via distinct pathways: Free fatty acids (FFAs) are unesterified lipids that function as energy substrates and signaling molecules. Elevated FFAs, a hallmark of metabolic dysregulation, activate inflammatory pathways (TLR4/NF-κB and NLRP3) in adipose and ovarian tissue, promoting neutrophil recruitment and platelet activation.^[29,30]^ IR-induced adipose dysfunction disrupts adipokine balance, further exacerbating inflammation. Elevated SII, reflecting this activation, then directly harms reproduction: Neutrophil-derived reactive oxygen species damages granulosa cells^[31]^; cytokines (IL-6, TNF-α) promote ovarian androgen excess and impair follicular maturation; oxidative stress compromises oocyte mitochondria^[20]^; and altered platelet function/lymphocyte activity may hinder endometrial receptivity and spiral artery remodeling.^[32-35]^ By quantifying the systemic inflammatory burden, SII provides an effective integrative clinical metric for understanding the potential connection between metabolic disturbances and reproductive dysfunction.

Limitations include the retrospective design (potential for unmeasured confounders like lifestyle/diet during treatment), single-timepoint measurements of metabolic/complete blood count parameters, and recruitment from a single fertility center potentially limiting generalizability. Future research should focus on mechanism validation and clinical translation. It is recommended to conduct prospective cohort studies to analyze the association between dynamic SII trajectories and IVF/ICSI outcomes. Furthermore, both basic and clinical interventional trials should be designed to test the hypothetical mechanistic pathways proposed in this study. For example, research could investigate the impact of metabolic interventions on SII and reproductive outcomes in PCOS animal models, or assess in randomized controlled trials whether anti-inflammatory therapy can reverse adverse reproductive outcomes in individuals with high SII, thereby directly validating the mediating role of SII.

## 5. Conclusion

In summary, this study establishes the SII as a novel and clinically accessible biomarker. It is the first to employ mediation analysis to reveal its potential mediating role between dyslipidemia and reduced IVF/ICSI live birth rates in women with PCOS. The integration of SII, calculated from routine blood counts, into clinical practice provides a feasible and cost-effective method to identify PCOS patients at heightened risk for IVF/ICSI failure. This enables a paradigm shift towards targeted, multi-faceted therapies that concurrently optimize metabolic health and mitigate inflammation (e.g., combining metformin/statin with low-dose aspirin or antioxidants). Such an approach holds significant promise for bridging the gap between metabolic dysfunction and reproductive success in PCOS.

## Acknowledgments

We want to express our thanks to all patients and their partners, nurses, doctors, and other medical staff in the Reproductive Center of Taizhou People’s Hospital affiliated with Nanjing Medical University for agreeing to participate in this study. We are also very grateful for the assistance of the proofreaders and editors.

## Author contributions

**Conceptualization:** Xiaofang Zhang, Hongshan Ge.

**Data curation:** Xiaofang Zhang.

**Writing – original draft:** Xiaofang Zhang, Ling Lin.

**Writing – review & editing:** Xiaofang Zhang, Hongshan Ge.
